# Congenital Hypertrophic Stenosis of the Pylorus

**Published:** 1904-10-15

**Authors:** 


### Hospital Clinics.

### CONGENITAL HYPERTROPHIC STENOSIS OF THE PYLORUS.

The recognition of this condition is a comparatively recent observation, but, now that it has been defined, it may be expected that more cases will be recorded. It would seem also highly probable that before the true nature of the pyloric obstruction was recognised a few cases had been described as examples of scirrhus of the pylorus in infants. Professor Osler[1] has discovered such a record in the reports of one of the medical societies of the United States as far back as 1788. In modern times Dr. J. Thomson, of Edinburgh, Dr. Cautley, and Mr. Clinton Dent have published valuable papers dealing with the subject. Dr. Cautley[2] has seen 10 cases since 1897. He points out that the condition is very apt to be overlooked unless a careful examination is made. Hence the vomiting, which is one of the symptoms in these cases, may be attributed to an error in diet or similar cause. The symptoms usually appear on the second or third week, but they may come on a few hours after birth, and in some instances they have been delayed for a month or more. Vomiting is the most important and most characteristic symptom. Usually two or three feeds are retained and are then vomited together so that the interval between successive attacks may be a fairly long one. Gradually the attacks become more frequent until even the smallest quantity of food is rejected. The vomit does not contain bile, but in the later stages mucus may be present. Constipation is another symptom of this condition, but though this is the rule it is not without exceptions. In typical cases the tongue is clean and the breath sweet. Examination of the abdomen may detect a wave of peristalsis passing along the gastric region from left to right, stopping momentarily at the pylorus and then continuing along the duodenum. If not present spontaneously it may be excited by the application of a cold finger to the epigastrium. Another physical fact of great importance is the detection of enlargement of the pylorus; this can be felt on careful palpation about half an inch to the right and three-quarters of an inch above the umbilicus. It is deeply seated and feels about the shape and size of a filbert. With these facts there will be progressive emaciation of the infant. The detection of the enlarged pylorus is of great importance as visible peristalsis may occur in simple dilatation of the stomach apart from stenosis. Possibly slight cases may be relieved by careful diet, rectal feeding, and by lavage and gavage, but in almost every case a diagnosis of congenital hypertrophic stenosis of the pylorus demands surgical interference. Mr. Clinton Dent[3] reports four cases in which he has successfully operated. He does not favour in this condition gastro-enterostomy, but advises dilatation of the pylorus, or pyloroplasty, with a decided preference for the latter. Dr. H. Willoughby Gardner[4] reports the case of an infant who was in good health until two months after birth, when it began to be troubled with vomiting followed by constipation and wasting; visible peristalsis of the stomach was observed, and an ill-defined tumour was detected in the region of the pylorus. He argues that the condition could not have been congenital, because until seven weeks of age the child was perfectly well and had gained fully 3 lbs. in weight. His suggestion is that, in consequence of ill-health on the part of the mother at this date, the breast-milk acquired an irritating quality. The first effect of this was diarrhœa, followed by spasm of the circular muscle of the pylorus, which in turn led to hypertrophy. It is noteworthy that though the vomiting was so persistent and the general condition of the child so unsatisfactory that at one time an operation was suggested, ultimately complete recovery was secured. This is attributed to the discontinuance of all foods which could possibly leave any undigested residue in the stomach and to the administration of nourishment in very small quantities. This case is obviously one of great importance and appears to support a suggestion made by Dr. Cautley in the paper already quoted, namely, that there are cases of pure pyloric spasm which may even lead to death from persistent vomiting, no hypertrophy of the pylorus being detected at the autopsy. In a case recorded by Dr. H. D. Rolleston,[5] there were two noteworthy features. In the first place in spite of repeated examinations no enlargement of the pylorus could be detected during life though at the post-mortem examination the hypertrophy was so great as almost to occlude the lumen of the sphincter. Secondly, it was observed that when six weeks old the child appeared to be in imminent danger of death from continued convulsions. The suggestion is that these were due to the absorption of toxic material from the stomach and that they may be compared to the attacks of tetany which sometimes accompany dilatation of the stomach in adults. In another instance, reported by Dr. Agnes Blackadder,[6] the only abnormal fact noted in the examination of the abdomen was the undue prominence of the superficial veins, no tumour was palpable, there were no signs of dilatation of the stomach, and the abdomen was not distended. Yet after death a sausage-shaped thickening of cartilaginous consistence and nearly an inch long was found in the pyloric region, and so greatly had this invaded the lumen of the bowel that water could not be forced through it from the stomach into the duodenum. All observers appear to agree that short of the severe cases which, unless promptly treated by operation, prove fatal in the early weeks or months of life there are minor degrees of the affection which cause symptoms that yield to suitable feeding. It has been suggested that some cases of troublesome vomiting and other gastric symptoms in adults may depend on a degree of congenital hypertrophy of the pylorus which has been aggravated by injudicious diet or other cause.

[1] Lancet, April 11, 1903. [2] British Journal of Children's Diseases, Jan. 1904. [3] Ibid. [4] Lancet, Jan. 10, 1903. [5] Brit. Med. Jour, Dec. 22, 1900. [6] Brit. Med. Jour., March 30, 1901.

